# Supplementary material for: Climate drives long-term change in Antarctic Silverfish along the western Antarctic Peninsula
Source: Commun Biol. 2022 Feb 3;5:104. doi: 10.1038/s42003-022-03042-3 (PMC8813954; doi:10.1038/s42003-022-03042-3)
Supplement: Supplementary file 2 — Supplementary information [file 42003_2022_3042_MOESM2_ESM.pdf]

Supplementary Information

Supplementary Figures

Supplementary Figure 1. Title: Palmer LTER study region during summer and winter conditions.

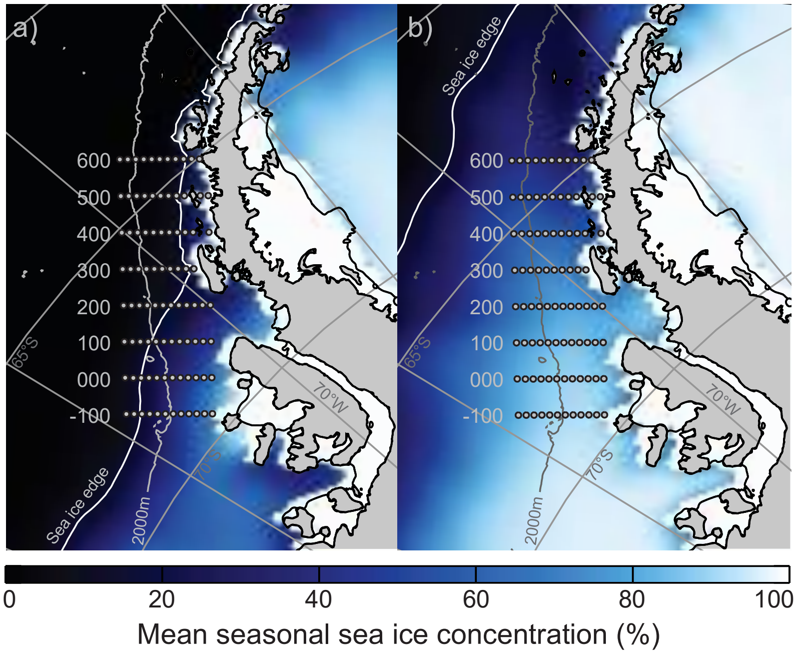

Description: **(a)** The Palmer LTER sampling grid with mean (1979 to 2020) sea ice concentration during the summer (DJF). The boundary between sea ice and open water is represented by the sea ice edge line. Stations are shown as circles and are spaced 20 km apart along grid lines (600 to -100) arranged perpendicular to the peninsula. Each grid line is spaced 100 km apart for a total sampling grid length of 700 km. The continental shelf boundary is also depicted by the 2000 m bathymetric contour line **(b)** The sampling grid with mean (1979 to 2020) sea ice concentrations shown during the winter (JJA).

Supplementary Figure 2. Title: Formulas of the final and base models.

a) Final model = glmmTMB(CPUE ~ sea surface temperature + 1-year lag sea ice advance + 1-year lag ASL MAM RCP + 1-year lag ASL MAM latitude + sea surface salinity + log(chlorophyll concentration) + AR1(year) + (1 | net tow coordinates), ziformula = all observations, family = tweedie(link = "log"))

b) Base model = glmmTMB(CPUE ~ sea surface temperature + sea surface salinity + log(chlorophyll concentration) + AR1(year) + (1 | net tow coordinates), ziformula = all observations, family = tweedie(link = "log"))

Description: **(a)** The structure for the final model, which was developed in R[1] using glmmTMB[2]. Acronyms not discussed previously are defined as: catch per unit effort (cpue; larvae 1000m^3), zero-inflation argument (ziformula), random effect (1 | …). **(b)** The structure of the base model, which was used to illustrate the influence of the ASL, SAM, and ENSO on model performance (Supplementary Table 3).

Supplementary Figure 3. Title: Predicted impact of chlorophyll concentration and salinity on Antarctic Silverfish larval abundance.

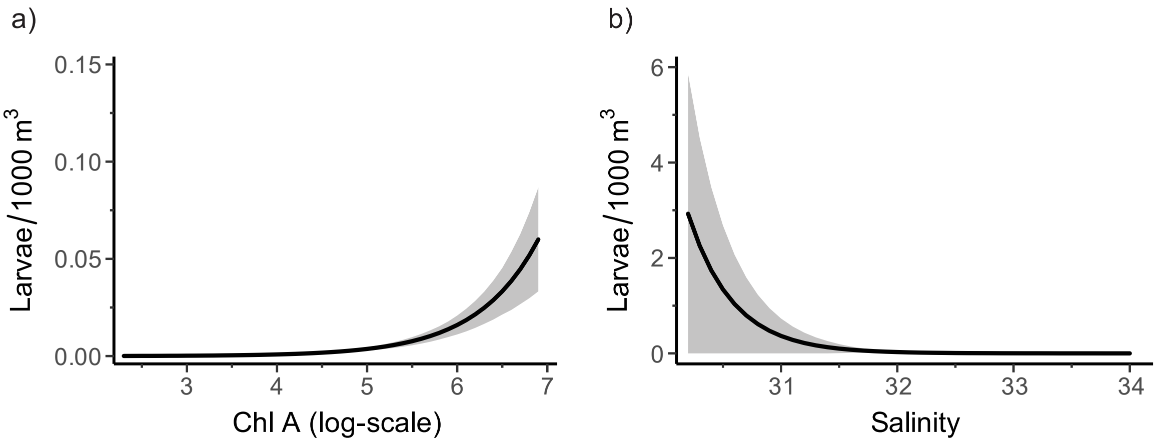

Description: Predicted impact (solid black lines) on larval Antarctic Silverfish abundance from **(a)** log-transformed chlorophyll concentration ($p < 0.001$) and **(b)** sea surface salinity ($p < 0.001$) from the final model (Supplementary Fig. 2a). The shaded regions represent the 95% prediction interval, which considers uncertainty from the fixed effects, zero-inflation, and random effects components of the final model (Supplementary Fig. 2a [3], see Materials and Methods).

Supplementary Figure 4. Title: Size distribution of Antarctic Silverfish larvae sampled by the Palmer LTER.

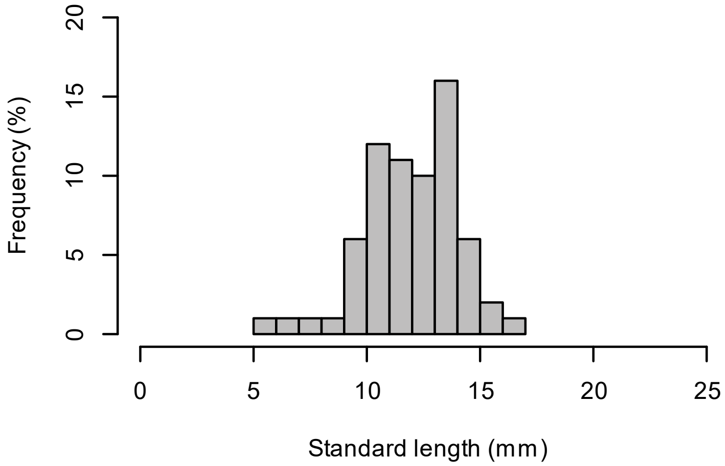

Description: Length distribution of Antarctic Silverfish larvae (n = 71) caught by the Palmer Antarctica Long-Term Ecological Research Program (Palmer LTER) off the western Antarctic Peninsula. The mean is 11.9 mm with a standard deviation of 2.1 mm. A subsample of larvae were randomly selected from the 7,093 fish used in this study to be measured. Note: the poor condition of the larvae prevented reliable measurements past the tenths place.

Supplementary Figure 5. Title: Residual diagnostic plots for the final model.

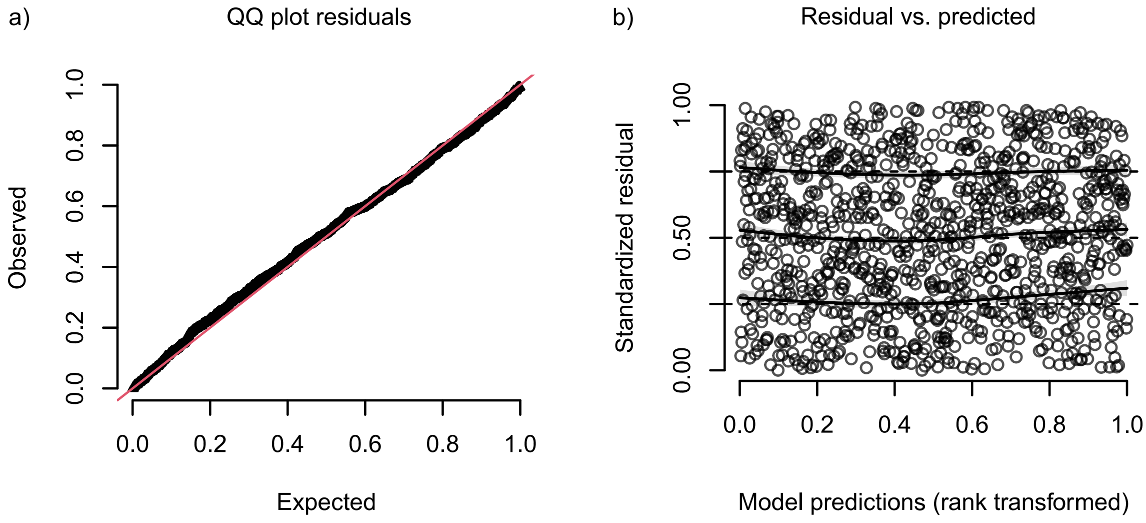

Description: **(a)** A uniform quantile-quantile (QQ) plot for a uniform distribution created from a DHARMa simulation output [4] of the final model (Supplementary Fig. 2a). **(b)** A plot comparing simulated quantile residuals, which have been standardized between 0 and 1 for ease of interpretation [4], to the rank-transformed predicted values from the fixed-effect component of the final model. Quantile regressions have been added (solid black lines) for the 0.25, 0.5, and 0.75 quantiles, which are represented by the dashed lines.

Supplementary Tables

Supplementary Table 1. Title: Results from the conditional component of the final model.

Variable	Estimate	Std. Error	Wald z	*p*-value
Sea surface temperature	-1.10	0.28	-4.00	< 0.001
Lagged sea ice advance	-0.08	0.01	-5.77	< 0.001
Lagged ASL MAM RCP	0.58	0.21	2.73	0.006
Lagged ASL MAM Lat.	0.26	0.09	2.83	0.005
Sea surface salinity	-2.43	0.44	-5.48	< 0.001
Log(Chlorophyll conc.)	1.47	0.30	4.92	< 0.001

Description: While several other variables were tested during the model development process (see Materials and Methods), only estimates for the parameters in the selected final model (Supplementary Fig. 2a) are displayed.

Supplementary Table 2. Title: Impact of temporal lagging strategy on model performance

Model structure	No. par	AIC	ΔAIC	Dev.
Final model (0-year lag)	8	1143.3	24.7	1117.2
Final model (1-year lag)	8	1118.6	0	1092.6
Final model (2-year lag)	8	1134.0	15.4	1108.0

Description: In each identical final model (Supplementary Fig. 2a), sea ice advance, ASL RCP, and ASL latitude are either not lagged (0-years), lagged by one year (1-year), or lagged by two years (2-year). Number of parameters (No. par), Akaike information criterion (AIC), the difference in AIC score (ΔAIC) between the given model and the best performing model, and the model deviance (Dev.) are displayed for comparison.

Supplementary Table 3. Title: Impact of the ASL, SAM, and ENSO on model performance

Model structure	No. par	AIC	ΔAIC	Dev.
Base (no clim. indices)	6	1128.8	10.2	1106.8
Base + annual ASL	7	1122.4	3.8	1098.4
Base + annual SAM	7	1127.9	9.3	1103.9
Base + annual MEI	7	1130.8	12.2	1106.8
Final model	8	1118.6	0	1092.6

Description: Annual indices of ASL, SAM, and ENSO (MEI) were each added to a base model (Supplementary Fig. 2b) to illustrate the relative impact of each climatic teleconnection on model performance. For this comparison, annual indices were used to partially account for differing seasonality of the three systems. The location of the ASL was also excluded from the base model. However, the final model (Supplementary Fig. 2a) contains ASL RCP and latitudinal location for the MAM period. Number of parameters (No. par), Akaike information criterion (AIC), the difference in AIC score (ΔAIC) between the given model and the best performing model, and the model deviance (Dev.) are displayed for comparison.

Supplemental References**:**

1. R Core Team. *R: A language and environment for statistical computing*. (R Foundation for Statistical Computing, 2020).
2. Brooks, M. E. *et al.* glmmTMB balances speed and flexibility among packages for zero-inflated generalized linear mixed modeling. *The R journal* **9**, 378–400 (2017).
3. Lüdecke, D. ggeffects: Tidy Data Frames of Marginal Effects from Regression Models. *JOSS* **3**, 772 (2018).
4. Florian, H. DHARMa: residual diagnostics for hierarchical (multi-level/mixed) regression models. http://florianhartig.github.io/DHARMa/ (2021).
