# Supplementary material for: Climate drives long-term change in Antarctic Silverfish along the western Antarctic Peninsula
Source: Commun Biol. 2022 Feb 3;5:104. doi: 10.1038/s42003-022-03042-3 (PMC8813954; doi:10.1038/s42003-022-03042-3)
Supplement: Supplementary file 3 — Reporting Summary [file 42003_2022_3042_MOESM3_ESM.pdf]

nature research

Corresponding author(s): Andrew D. Corso
Last updated by author(s): 16 November 2021

Reporting Summary

Nature Research wishes to improve the reproducibility of the work that we publish. This form provides structure for consistency and transparency in reporting. For further information on Nature Research policies, see our Editorial Policies and the Editorial Policy Checklist.

Statistics

For all statistical analyses, confirm that the following items are present in the figure legend, table legend, main text, or Methods section.

n/a	Confirmed	
☐	☒	The exact sample size (n) for each experimental group/condition, given as a discrete number and unit of measurement
☐	☒	A statement on whether measurements were taken from distinct samples or whether the same sample was measured repeatedly
☐	☒	The statistical test(s) used AND whether they are one- or two-sided<br>*Only common tests should be described solely by name; describe more complex techniques in the Methods section.*
☐	☒	A description of all covariates tested
☐	☒	A description of any assumptions or corrections, such as tests of normality and adjustment for multiple comparisons
☐	☒	A full description of the statistical parameters including central tendency (e.g. means) or other basic estimates (e.g. regression coefficient) AND variation (e.g. standard deviation) or associated estimates of uncertainty (e.g. confidence intervals)
☐	☒	For null hypothesis testing, the test statistic (e.g. F, t, r) with confidence intervals, effect sizes, degrees of freedom and P value noted<br>*Give P values as exact values whenever suitable.*
☒	☐	For Bayesian analysis, information on the choice of priors and Markov chain Monte Carlo settings
☐	☒	For hierarchical and complex designs, identification of the appropriate level for tests and full reporting of outcomes
☒	☐	Estimates of effect sizes (e.g. Cohen's d, Pearson's r), indicating how they were calculated

Our web collection on statistics for biologists contains articles on many of the points above.

Software and code

Policy information about availability of computer code

Data collection	Monthly sea ice concentration anomalies are derived from the Scanning Multi-channel Microwave Radiometer-Special Sensor Microwave/ Imager (SMMR/SSM/I) satellite time series based on the Goddard Space Flight Center (GSFC) Bootstrap algorithm. The sea ice concentration data are gridded to 25 km and are provided by the EOS Distributed Active Archive Center (DAAC) at the National Snow and Ice Data Center (NSIDC, University of Colorado at Boulder, http://nsidc.org). The numerically-analyzed monthly 10-m height winds and sea-level pressure anomalies are from the fifth generation European Centre for Medium Range Weather Forecasts (ECMWF) Reanalysis (ERA-5) and are provided by the Climate Data Store (CDS, https://cds.climate.copernicus.eu/).<br><br>Sea surface temperatures for the Palmer LTER study region were determined by the NOAA optimal interpolation (OI) sea surface temperature analysis (version Reyn_SmithOI.v2) using in situ and satellite sea surface temperatures, plus sea surface temperatures simulated by sea ice cover.<br><br>We considered ocean temperatures and salinity from near the sea surface (5 m depth), the bottom of the tow depth (120 m), and averaged from 120 m to the surface. All temperature and salinity measurements were collected using CTD casts that were spatially and temporally paired with net tows. Discrete measurements of Chlorophyll a concentration measured in water collected in CTD casts were integrated to 100 m and also paired spatially and temporally with net tows. Sea ice variables considered were derived from satellite imagery (Scanning Multichannel Microwave Radiometer and Special Sensor Microwave/Imager; SMMR-SSM/I) and included duration, extent, day of retreat, and day of advance.<br><br>Annual indices of climatic teleconnections (e.g., ENSO, SAM, and ASL) were included in the model. The ENSO index is based on sea surface temperatures (referred to as the multivariate ENSO index [MEI]; http://www.esrl.noaa.gov/psd/people/klaus.wolterlter/MEI/) and the SAM (http://www.antarctica.ac.uk/met/gjma/sam.html) index is based on sea level pressure. These climate indices are seasonally adjusted. The RCP and latitudinal location of the ASL were obtained from Hosking et al 2020. The RCPs from all four seasons (DJF, MAM, JJA, and SON) were evaluated.

Data analysis	Zero-inflated Generalized Linear Mixed-Effects Models (GLMMs) and associated analyses were developed in the R software environment. All associated packages used in the study (e.g., glmmTMB, ggeffects, car, ggplot2, etc.) are freely available online.

For manuscripts utilizing custom algorithms or software that are central to the research but not yet described in published literature, software must be made available to editors and reviewers. We strongly encourage code deposition in a community repository (e.g. GitHub). See the Nature Research guidelines for submitting code & software for further information.

Data

Policy information about availability of data

All manuscripts must include a data availability statement. This statement should provide the following information, where applicable:

- Accession codes, unique identifiers, or web links for publicly available datasets
- A list of figures that have associated raw data
- A description of any restrictions on data availability

The Antarctic Silverfish data that support the findings in this study are archived in the VIMS Nunnally Ichthyology Collection and are publicly available on the VIMS Specify web portal (https://www.vims.edu/research/facilities/fishcollection/search_collection/index.php). The associated Palmer LTER environmental variables are available online from the Palmer LTER web portal. See https://pallter.marine.rutgers.edu for more information.

Field-specific reporting

Please select the one below that is the best fit for your research. If you are not sure, read the appropriate sections before making your selection.

☐ Life sciences ☐ Behavioural & social sciences ☒ Ecological, evolutionary & environmental sciences

For a reference copy of the document with all sections, see nature.com/documents/nr-reporting-summary-flat.pdf

Ecological, evolutionary & environmental sciences study design

All studies must disclose on these points even when the disclosure is negative.

Study description	The density (abundance divided by tow volume [in units of 1000 m3]) of larval Antarctic Silverfish for each pelagic net tow from 1993 – 2017 was modeled against environmental variables. Zero-inflated Generalized Linear Mixed-Effects Models (GLMMs) following a Tweedie distribution were developed in R and used to model the relationships between environmental variables and larval abundance.
Research sample	Larval fishes were collected annually from 1993 through 2017 as part of the Palmer Antarctica Long-Term Ecological Research (Palmer LTER) program. Scientists on Palmer LTER cruises collect multidisciplinary data in a fixed-sampling grid in the Bellingshausen Sea along the western Antarctic Peninsula (WAP). Zooplankton and larval fishes are sampled each year during austral summer (January to February) using a 2-m2 frame Metro net (700-µm mesh) towed to ~120m depth.
Sampling strategy	No sample size calculation was performed, all samples were initially included in this study.
Data collection	Larval fishes were collected annually from 1993 through 2017 as part of the Palmer Antarctica Long-Term Ecological Research (Palmer LTER) program. Scientists on Palmer LTER cruises collect multidisciplinary data in a fixed-sampling grid in the Bellingshausen Sea along the western Antarctic Peninsula (WAP). Zooplankton and larval fishes are sampled each year during austral summer (January to February) using a 2-m2 frame Metro net (700-µm mesh) towed to ~120m depth.
Timing and spatial scale	Larvae are then sampled annually by the Palmer LTER during January and February.
Data exclusions	The total sample size of larval Antarctic Silverfish collected from 1993 - 2017 by the Palmer LTER is 10,644 individuals. However, larvae were excluded that a) were opportunistically sampled at "negative" Grid Stations (0 to -60) of the Palmer LTER grid in Marguerite Bay (see https://pal.lternet.edu/research/sampling-grid) or b) did not have the necessary environmental data associated with the collection event (e.g., sea surface temperature or salinity). After this initial exclusion, the total sample size was reduced to 7,086 individuals. This sample (n = 7,086) was used in the final model.
Reproducibility	No experiments were conducted in this study.
Randomization	No experiments were conducted in this study and randomization was not required.
Blinding	No experiments were conducted in this study and blinding was not necessary.

Did the study involve field work? ☒ Yes ☐ No

Field work, collection and transport

Field conditions	Field work was conducted near the western Antarctic Peninsula. For more information on the regional conditions, please see https://pal.lternet.edu/about/overview/site-description.
Location	All field work was conducted on the sampling grid of the Palmer Antarctica Long-Term Ecological Research Program, see https://pal.lternet.edu/research/sampling-grid.

Access & import/export	All field work, collection, and transport was performed following the requirements and principles established by the United States Antarctic Program (USAP) Environmental, Health, and Safety (EH&S) Division. See https://www.usap.gov/environmental/?m=1 for more information.
Disturbance	Pelagic net tows and the collection of zooplankton was a minor disturbance to the western Antarctic Peninsula pelagic ecosystem. These tows were minimized when possible and strictly performed on the established Palmer LTER sampling grid.

Reporting for specific materials, systems and methods

We require information from authors about some types of materials, experimental systems and methods used in many studies. Here, indicate whether each material, system or method listed is relevant to your study. If you are not sure if a list item applies to your research, read the appropriate section before selecting a response.

Materials & experimental systems

n/a	Involved in the study
☒	☐ Antibodies
☒	☐ Eukaryotic cell lines
☒	☐ Palaeontology and archaeology
☐	☒ Animals and other organisms
☒	☐ Human research participants
☒	☐ Clinical data
☒	☐ Dual use research of concern

Methods

n/a	Involved in the study
☒	☐ ChIP-seq
☒	☐ Flow cytometry
☒	☐ MRI-based neuroimaging

Animals and other organisms

Policy information about studies involving animals; ARRIVE guidelines recommended for reporting animal research

Laboratory animals	The study did not involve laboratory animals.
Wild animals	Larval Antarctic Silverfish (Pleuragramma antarctica) were incidentally captured as bycatch during pelagic net tows conducted by the Palmer Antarctica Long-Term Ecological Research (Palmer LTER) Program from 1993 – 2017. The larval fishes are predominantly year-0 and cannot feasibly be sexed. Following capture, larvae were immediately separated from other zooplankton and preserved in a formaldehyde solution.
Field-collected samples	The study did not involve laboratory work with samples collected from the field.
Ethics oversight	No ethical approval or guidance was required as sampling was targeted for the collection of zooplankton.

Note that full information on the approval of the study protocol must also be provided in the manuscript.
